# Supplementary material for: Are carbon emissions trading and green financial instruments synergistic? -Comprehensive quantitative research based on content analysis
Source: PLoS One. 2024 Mar 7;19(3):e0298601. doi: 10.1371/journal.pone.0298601 (PMC10919601; doi:10.1371/journal.pone.0298601)
Supplement: S1 Data — (ZIP) [file pone.0298601.s001.zip › Data/地级市Z_F工作报告.zip!/╕ú╜¿/┴·╤╥╩╨/└┤╫╘╣1⁄2╓┌║┼í╛┬φ┐╦╩2╛▌═°í┐_2005.docx]

­——2005年2月26日在龙岩市第二届人民代表大会第五次会议上

　　龙岩市人民政府市长 刘赐贵

各位代表：

　　现在，我代表市人民政府向大会作政府工作报告，请予审议，并请市政协各位委员和其他列席人员提出意见。

　　一、2004年工作回顾

　　2004年，我市各级政府坚持以邓小平理论和“三个代表”重要思想为指导，认真贯彻党的十六大和十六届三中、四中全会精神，树立和落实科学发展观，围绕建设海峡西岸经济区，积极落实省委、省政府及市委的决策部署，通过全市上下的共同努力，克服了发展中遇到的各种不利影响和困难，较好地完成了市二届人大四次会议确定的工作任务，10项预期指标全部超额完成。初步核算，全市完成地区生产总值348.3亿元，按可比价格计算，增长10.5%，其中第一产业73.7亿元、增长6%，第二产业153.7亿元、增长12.6%，第三产业120.9亿元、增长10.8%；全社会固定资产投资91.8亿元，增长22.4%；财政总收入47.2亿元（不含基金），增长18.1%，其中地方级收入17亿元（不含基金），增长18.5%；外贸出口2.1亿美元，增长117%；实际利用外资9107万美元，增长96.3%；社会消费品零售总额77.6亿元，增长15%；城镇居民人均可支配收入1.02万元，增长12.3%；农民人均纯收入3769元，增长11.7%；城镇登记失业率6%；人口自然增长率5.8‰。

　　项目工作成效突出。“三个一百”项目中，138个新增长点项目新增产值38.5亿元，拉动GDP增长3.4个百分点；160个投资项目完成投资64.8亿元，占全社会固定资产投资70.6%，其中赣龙铁路、龙长高速公路、坑口火电厂等37个在建、新开工重点项目完成投资36.3亿元，增长18.9%。全市新开工投资千万元以上项目168个，永定兴鑫水泥一期、卓鹰钢铁一期、马坑铁矿一期、三德水泥二期、卫东工业除尘器生产线、长汀纺织基地、紫金铜矿开发、龙工齿轮生产线等一批重大项目顺利实施；8个工业园区新上企业102家、新投产企业60家。全市实现全部工业总产值312亿元，增长15.7%；规模以上工业总产值213.6亿元，增长20.6%；新增规模以上工业企业119家，累计461家；新增产值超亿元工业企业12家，累计31家；规模以上工业经济效益综合指数212.3，居全省首位。全市投入国企改制资金1.27亿元，新完成16家国有工业和商贸企业产权置换、4551名职工身份置换；国有工业和商贸企业累计改制面分别为82.8%和71.3%，职工身份置换面分别为94.5%和84.1%。开展大型招商活动8场。全市新登记外资企业户数、注册资本、外方认缴资金分别增长83%、166.3%和185.6%，新登记个体工商户数、从业人员、注册资本分别增长52%、18.6%和26.8%。全市非国有经济占城镇以上固定资产投资和规模以上工业产值的比重分别提高22.7和2.2个百分点，民营企业出口比重提高12个百分点。

　　“三农”工作继续加强。认真落实扶持粮食生产、集体林权制度改革、农村低保、税费改革、劳动力培训转移等支农政策措施，全市发放粮食生产补助资金456万元，粮食产量增长3.9%；集体林权制度改革完成1408个村、1377.2万亩，分别占总数的80.4%和93.2%，造林更新完成省计划的110%；免征农业税4500万元；4.49万户、11.6万人享受农村低保，累计发放低保金4896万元；新增转移农村劳动力6.2万人，人均外出劳务收入8930元、增长19.6%。全市收购烤烟61.5万担，增加11.3万担；生猪出栏467.7万头，增加60.2万头，畜牧业占农林牧渔业产值比重提高6.5个百分点。新发展农业专业合作组织和农产品行业协会15个；新增国家级农业产业化认证企业1家、省级2家、绿色食品标志使用权4个、无公害农产品认证8个。完成农村公路路面硬化1843公里、防洪堤建设11.4公里、水库除险加固34座、中低产田改造2.5万亩、农田节水灌溉6.1万亩、水土流失治理17.5万亩和10个乡镇、157个村的通水工程。继续开展了农村合作医疗试点。扶持14个乡镇卫生院配备医疗设施，培训医护骨干370名。完成102个村铺设广电光缆。选派477名干部驻村任职，继续挂钩帮扶190个村、2100户贫困户和低收入农户，为2万农户发放“小额信贷”资金4500万元，“造福工程”搬迁3699人。

　　城镇建设稳步推进。开展了龙岩中心城市总体规划修编，完成了新区城市规划设计，中心城市控规覆盖面达80%。城区面积扩大到29平方公里，新建道路5.6公里、污水管网10.7公里、园林绿地22.9公顷。龙腾南路开发区段一期全线贯通，龙川路、解放南路改造完成，北二环路一期、龙腾路二期、莲西路二期、北环路二期、龙腾北路、双洋路、石埠立交接线工程、铁山立交桥正在建设，龙岩图书馆、黄竹坑垃圾处理场等项目投入使用。对一批市政公用设施的建设、管理实行了市场化运作。县（市）城关、重点镇和示范新村建设稳步推进。第三产业较快发展，旅游接待326万人次，总收入14.7亿元，分别增长16.4%和20.4%；全市限额以上贸易业零售总额增长33.6%，新增连锁超市10家，闽西粮油饲料城开工建设；商品房销售面积70.4万平方米、销售额10.8亿元,分别增长44.2%和32.7%；新建社区服务中心115个；金融、保险、电信、运输等服务业保持较快发展。

　　社会发展持续协调。计生工作连续14年完成省下达人口控制计划。国土资源保护连续5年实现耕地占补平衡，“青山挂白”治理取得阶段性成效，国有土地使用权和矿业权出让工作更加规范有序。全市落实生态公益林管护面积656.7万亩，漳平天台山和永定王寿山被评为国家级森林公园。投入9000万元完成了九龙江流域龙津河段两岸1228家生猪养殖场搬迁。累计投入1.2亿元完成了全市116家水泥企业的粉尘治理，中心城市空气质量达到良以上天数264天，比整治前增加79天。全市“双高普九”工作扎实推进,新罗区顺利通过省级评估验收；农村中小学危房改造工程第二期第一批项目基本完成；高考万人口上本科线人数32.8人,比增5.8%；龙岩学院、闽西职业技术学院正式揭牌，市行政学院、经济干校与市委党校合并办学。中心城市顺利通过省级卫生城市评估,新组建了市疾病预防控制中心和卫生监督所，市中心血站、中医院门诊大楼、疾病预防控制中心实验大楼、广电大楼、体育公园、体育大厦等基础设施项目建设顺利。竞技体育成绩突出，石智勇荣获第28届雅典奥运会男子62公斤级举重金牌，为全国人民争得了荣誉。古田会议旧址群维修全面完成，永定土楼申报“世遗”被正式受理，连城县被评为“中国首批优秀旅游县”。全市新增城镇就业2万人，下岗失业人员实现再就业1.79万人，长汀县就业和再就业工作受到国务院表彰；养老、失业、医疗、工伤、生育保险以及城乡低保等社会保障体系进一步完善；提高了企业退休人员养老金补助标准；出台了《龙岩市优待老年人若干规定》；治理餐桌污染、建设“食品放心工程”等11件为民办实事项目如期完成。外事侨务、民政、民族宗教、国防动员、人防、民兵预备役、档案、地方志、妇女儿童、残疾人等工作取得新成效。

　　投资环境不断优化。应对禽流感、干旱和电力、煤炭、运力紧张等困难问题取得较好成效。行政审批制度改革继续深化，对市、区278项审批事项实行了“一审一核制”，行政服务中心网络审批平台7月1日正式运行，窗口审批事项立即办理率提高49个百分点。市项目中心较好地发挥了项目储备、推介、咨询洽谈的平台作用。财政、税务、金融、电力、土地、技术监督、科技、人才等部门的支持服务作用增强。全市扶持企业、项目财政性资金3.6亿元，其中市本级7029万元；出口退税2.38亿元，增长569%；金融贷款增长16.2%；全社会用电增长10.9%；新增中国名牌产品1个、省名牌产品3个；龙净环保企业技术中心成为全国同行业第1家国家级企业技术中心，卫东环保、西湖环保、卓越新能源、华锐刀具4个企业被认定为省级高新技术企业。

　　民主法制更加健全。坚持依法治市、依法行政，自觉对市人大及其常委会负责并报告工作，认真执行人大决议，主动接受人大的法律监督和工作监督。加强与政协和各民主党派、工商联、工会、共青团、妇联、科技智囊团、无党派人士、老同志及社会各界的联系，主动通报情况，接受监督。认真配合开展人大代表、政协委员的视察、检查、调研活动，143件人大代表建议和220件政协委员提案已全部办结。认真贯彻实施《行政许可法》，废止17份与法律法规不相符的文件，清理了11个不具备行政许可资格的机构和组织。积极开展创建“平安龙岩”活动，全市刑事发案率下降1.5个百分点，破案率上升7.1个百分点，居民满意率95.6%，居全省第三位。加强安全生产监管，全面落实责任制，深入开展煤矿和其它行业安全生产专项整治并取得成效。“整规”工作加强，市场经济秩序正常。全市清理建设领域拖欠农民工工资9517万元。加强行政监察和审计监督，推进廉政建设、效能建设、政务公开和纠风工作，全市行政监察机关立案和处理监察对象违纪问题206起，对174人次进行了效能告诫。

　　各位代表，过去一年所取得的成绩，是省委、省政府和市委正确领导的结果，是全市人民聚精会神搞建设、一心一意谋发展的结果，是方方面面团结协作、相互配合、大力支持的结果。在此，我代表市人民政府向积极参与经济社会发展的广大干部群众，向热情支持政府工作的人大代表、政协委员和各民主党派、工商联、群团组织、无党派人士、离退休老同志，向中央和省驻岩机构、驻岩部队、公安干警和武警官兵，向所有关心支持龙岩发展的海内外朋友们，表示衷心的感谢和崇高的敬意！

　　在肯定成绩的同时，我们也清醒地看到，当前经济社会发展中还存在不少问题，主要是：发展水平总体上还比较滞后，经济总量偏小，与全省平均水平和沿海发达地区相比有一定差距；一些地方和干部用科学发展观指导工作的能力有待进一步提高，工作不平衡导致发展不平衡的问题比较突出；项目质量总体不高，非资源型项目、产业链延伸项目和科技含量高、投资规模大的项目比较缺乏；中心城市集聚辐射能力不强；乡村财政比较困难，城乡社会保障能力较低；一些地方环境污染和脏、乱、差现象不同程度存在；安全生产和社会稳定还有不少薄弱环节；少数机关部门和工作人员作风不实，缺乏责任意识、公仆意识、大局意识、创新意识，存在消极腐败、铺张浪费现象。我们将进一步采取措施解决好这些问题。

　　二、2005年工作安排

　　2005年，是完成“十五”计划和市委提出的全面建设小康社会三年打基础、衔接“十一五”发展的重要一年。政府工作的总体思路是：认真贯彻党的十六大和十六届三中、四中全会精神，按照中央经济工作会议和省、市委的部署，树立和落实科学发展观，围绕建设海峡西岸经济区，继续坚定不移实施项目带动战略和扩大对外开放，把龙岩建成闽粤赣边联结沿海、拓展腹地的生态型经济枢纽，使闽西成为海峡西岸经济区的重要增长极。协调推进物质文明、政治文明、精神文明建设，扎实构建社会主义和谐社会。

　　主要预期目标：地区生产总值增长10.5%，其中第一产业增长4.5%，第二产业增长13.5%，第三产业增长10.5%；全社会固定资产投资增长13.6%；财政总收入、地方财政收入增长10%，其中市本级财政总收入增长10.5%、地方财政收入增长12%；外贸出口增长15%；实际利用外资增长25%；社会消费品零售总额增长12%；城镇居民人均可支配收入增长6%；农民人均纯收入增长5%；城镇登记失业率控制在5.8%以内；人口自然增长率控制在6‰以内。

　　围绕上述思路和目标，重点抓好六项工作：

　　（一）坚定不移实施项目带动战略，不断促进产业发展

　　深化项目带动战略地位和作用的认识。坚持资源型产业与非资源型产业并举，在抓好资源型接续产业的同时，大力发展非资源型产业；坚持基础性项目与生产性项目并举，在继续重视基础性项目尤其是交通项目的同时，大力发展生产性项目；牢牢把握符合国家产业政策、符合可持续发展要求和符合新的经济增长点、新的财税点、新的就业点的项目选择原则，继续从向上争取、利用外资和区域协作、激活民资、现有企业延伸做大等渠道推进项目，努力形成抓项目和抓发展的强大合力。

　　重点抓好“三个一百”项目。初步安排新增长点项目143项，年新增产值45.5亿元；投资项目168项，总投资331亿元，年度投资88.1亿元；储备项目115项，总投资391.3亿元。在投资项目中，实施重点项目64项，年度投资62.6亿元，其中在建重点项目29项，年度投资53.7亿元；预备新开工项目22项，年度投资8.9亿元；重点前期工作项目13项，总投资290.7亿元。继续对“三个一百”项目和重点项目实行目标管理，强化领导挂钩责任制、定期协调例会制以及督查联动机制，及时有效解决项目推进中遇到的困难问题，确保龙长高速公路、赣龙铁路、坑口火电厂、白沙水库电站、汀州水电站、闽西粮油饲料城、省运会主场馆等项目完成年度建设任务，永武高速公路、龙厦铁路、漳永支线高速公路、汀江防洪三期等项目前期工作实现计划目标。

　　继续加强招商引资工作。加强以商引商、小型招商、产业招商，借助外事、侨台、区域协作、对口帮扶、友好城市、投资协会、商会、政府投资与发展顾问、网络等渠道扩大招商。继续在港澳台地区、东南亚国家和珠三角、长三角发达地区以及本省沿海地区开展重点招商，精心组织参加“6·18”、“9·8”等招商活动，举办第二届“投资龙岩”项目洽谈会。落实招商引资责任制，开展评选市经济发展突出贡献奖活动，形成政府招商、部门招商、企业招商、民间招商的合力。加强在谈项目和签约项目的跟踪服务，确保实际利用外资1亿美元以上、实到市外资金25亿元以上。

　　发挥民营经济在发展中的积极作用。按照“非禁即入”的原则，让民营企业参与投资更多的领域、更多的项目。做好污水处理、园林管养、道路洒水、户外广告、液化气经营、公交、农村自来水等公用项目市场化，研究配套政策，吸引民资进入。加强证照、信息、人才、中介、贷款担保服务，为民营企业在投资、融资、税收、土地等方面创造公平竞争环境。提升民营经济竞争力，使民营经济成为调整经济结构、优化生产力布局、增加社会就业、繁荣城乡经济的生力军。

　　不断加强投资环境建设。落实《龙岩市促进投资与企业发展的规定》及其10个配套实施意见，继续实行出口奖励政策。完善各个工业园区的基础设施、服务机制和招商政策，为项目落地提供更有吸引力的综合平台。全面落实项目审批改核准或备案的具体措施，建立部门内部行政许可职能相对集中的工作机制，进一步简化审批环节，创新审批方式，缩短审批时间。加强项目用地、用工、用电、用水服务。依法征税，认真落实税收优惠政策，做好出口退税工作；增收节支，加强资金调度，加大财政对项目的支持力度。

　　加强重大项目开发。认真组织开展生态型经济枢纽建设纲要和“十一五”规划编制，加强产业发展、基础设施、开放协作、生态环境、社会保障、和谐社会等支撑体系重大项目的研究，开发一批重大生产性项目、龙头企业配套项目、产业集群项目、科技成果转化项目。依托现有企业、投资商、政府部门和科研院所深化项目开发，推进项目库建设和市、县项目资源共享，加强项目推介、咨询与跟踪服务，促进项目生成落地。

　　（二）加强工业组织领导，确保较快较好增长

　　认真落实烟草、冶金、环保、汽车、工程机械、纺织服装、建材、能源、化工等重点产业、重点行业发展规划，以项目为依托，实现全部工业总产值380亿元、增长14.5%，其中规模以上工业总产值270亿元、增长21%；新增规模以上工业企业120家以上。

　　突出抓好150个工业新增长点项目。重点加快龙工齿轮生产线、龙佳精密铸锻件、龙马机械公司轻卡、力菲克药业二期等非资源型重大项目的实施；继续抓好龙烟技改、国产实业水泥、漳平大深水泥和红狮水泥、紫金铜矿开发、紫金铜板带材生产线、卓鹰钢铁二期、兴鑫水泥二期、马坑铁矿二期、龙宝无烟煤加工二期、高岭土等资源精深加工项目的建设。

　　加强重要原材料和生产要素协调服务。依法加快重点矿井建设，加强煤炭供应调控，努力满足企业生产需求。加快供配电工程建设，鼓励有条件的企业实施余热发电，积极向上争取电量，促进节约高效用电。落实道路运输规费调整等相关政策，加强铁路运输品种、车皮调控，增强运力保障。继续定期组织银企洽谈会，积极争取金融支持，加快福建煤电、龙工等有条件的企业上市。厉行节约原则，坚持合理、有效利用土地，确保工业项目用地。

　　加快推进企业技术创新。抓好30个重点技改项目、20个技改前期项目和100个新技术、新产品开发、电子信息应用项目。加强科技服务，支持企业建立市级以上技术中心，申报国家、省级技术创新项目，促进企业与科研院所开展技术合作。支持企业争创省级以上名牌或商标。

　　基本完成国有企业改革。按照优先置换职工身份的原则，继续抓好市属国有企业改革。完成市属7大行业公司改制，理顺行业管理服务体制。落实《市属国有企业改革预留资金筹措和管理规定》，解决好改制企业遗留的社保、负资产等问题。组建市国有资产监督管理委员会，加强对国有资产授权公司及国有企业（含参控股企业）的监管和考核，实现国有资产安全运行和保值增值。

　　（三）继续加强“三农”工作，加快农村经济发展

　　进一步落实支农政策措施。全面完成集体林权制度改革，深化农村税费、粮食流通体制、土地流转、小型水利设施产权改革，启动农村信用社管理体制改革。推广“公司+协会+农户”的经营模式，积极发展农业专业合作组织和农产品行业协会，力争带动农户30%以上。继续加强劳动力培训转移，完成技能培训2万人，新增转移6万人。健全帮扶薄弱村工作机制，推广村账委托代理制，有效化解乡村债务，抓好撤乡并镇试点。

　　提高农业综合生产能力。继续抓好粮食生产，积极发展烤烟、早熟水果、优质茶、食用菌、反季节蔬菜、花卉、水产等优势特色产业，完成30万亩造林任务。落实养殖业发展规划，推行“猪沼果（菜）”等生态养殖模式。继续鼓励现有龙头企业发展壮大，重点培育新的龙头企业，抓好森宝生猪、新罗国葳笋豆制品、长汀盼盼食品、连城地瓜干加工等项目，力争农产品加工率达到35%以上。加快建设绿色、无公害农产品生产基地，大力推行标准化生产，力争绿标产品达到30个以上，无公害农产品认证达到75个以上。加强村级农技员队伍建设，抓好与高等院校、科研机构的农业科技协作，强化动植物疫病监测、防治，提高农业科技创新能力。

　　加强农村基础设施建设。继续实施农村卫生院“百院建设、千人培训”计划，加强村卫生所人员业务培训并给予适当补助，推进新罗区新型农村合作医疗试点。继续扶持农村公路建设，完成所有县通乡镇和700公里乡镇通村公路路面硬化。继续抓好农村水利“六千”工程，新建小型水库5座、江堤10公里、标准化渠道200公里，新增农田节水灌溉5万亩，完成2座中型和31座小型水库除险加固，整治河道22.6公里，治理水土流失18万亩，解决262个村通水。完成60个村和141个50户以上自然村通广播电视，新增联网入户3万户，实现69%的村通光缆。抓好129个省、市挂钩贫困村的整体脱贫示范工作，实施造福工程搬迁3000人。

　　（四）加快发展第三产业，提升层次壮大总量

　　大力发展旅游产业。做好以古田会议旧址群为主的“全国红色经典旅游景区”、永定土楼、冠豸山等重点景区的建设和管理，提高旅游接待能力和服务质量。开展以厦漳龙为重点的闽粤赣13市无障碍区域旅游协作，办好市第三届旅游节，拓展珠三角、长三角和闽南金三角以及港澳台、日本、东南亚市场。开发具有区域特色的旅游商品，加强旅游人才培养，加大旅游资源的招商开发力度。

　　继续发展商贸和房地产业。加快闽西粮油饲料城、闽西交易城、中山路等商业市场、商业街区的建设和完善。依托大型超市和商场，发展电子商务、物流配送、连锁经营。积极引导房地产业健康有序发展，加强普通住宅、高层住宅、生活小区、经济适用房的规划建设，严格执行设计标准。

　　重视发展新兴服务业。加快发展物流、技术研发、商务信息、资产评估、文化娱乐、人才、法律、金融、保险、电信等服务业，积极发展社区服务业。完善土地、林权、矿业权等产权市场。加强各类中介机构和服务业的培育、监管，规范服务行为。

　　（五）加快中心城市发展，增强辐射带动能力

　　做大支柱产业。重视抓好市、区两级的骨干企业、品牌企业的发展。加快市、区两级工业园区建设，抓好龙岩经济技术开发区东肖片区建设和东山片区规划开发，推进新罗民营科技园区张白土片区和东宝山片区发展。

　　完善基础设施。继续滚动实施2003—2007年100个城建项目，重点实施龙腾路二期、双洋路、北二环路一期、龙腾南路、铁山立交等道路项目，城区供水管网改造、污水管网支管一期、登高西路污水管道、垃圾中转站等公用设施项目，完成九一路、凤凰路路面重铺。推进第二水源项目前期工作。增加出租车投放量，分批更换公交车辆，建设停靠站点，加大运行密度，延伸运行线路。抓好宾馆、酒店改造和新建，提高接待能力。

　　加强规范管理。继续做好总规修编，完成畅通工程、商业网点、公交、停车场等专项规划编制，中心城市控规覆盖率达到85%以上。加强规划控制和城建监察，探索城市规划、土地监察执法权委托下移。按照权责利相统一和功能齐全、运作高效的原则，下放环卫管理权，建立城市早夜市临时摊点、城乡结合部环境卫生、户外广告、建筑工地等市容市貌长效管理机制。加强中心城市村级自用地管理。稳步推进镇改街、村改居试点。

　　改善环境质量。以争创省级园林城市为目标，巩固大气污染治理成果，推进“五小”企业、燃煤锅炉、建筑和道路扬尘、机动车尾气、油烟、噪音治理。继续抓好龙津河整治，全面治理九龙江沿河两岸养殖场等污染源。制订并实施中心城市一重山范围内所有污染企业、矿山的分步搬迁、关闭计划。美化、亮化登高山、虎岭山等城市山体。

　　（六）加大统筹力度，促进协调发展

　　充分发挥人才对发展的支撑作用。发挥现有大中专学校和各类培训机构的作用，加强人才培养。健全人才选拔任用机制和聚才引智机制，做好人才储备工作。落实技术、知识、管理等要素参与分配的政策，改善工资福利、社会保障、住房等条件，留住现有人才。完善人才市场服务体系，推进博士后站点等载体建设，加强与院士、专家、学者的联系，努力吸引省内外人才来岩服务或参与发展。

　　促进可持续发展。大力发展循环经济，积极推广清洁生产、节能技术和废旧资源回收利用，淘汰浪费资源、污染环境的工艺、设备和产品，提高资源利用效率和产出效益，严禁新上污染企业。强化计生目标管理责任制，加强村、居基层基础管理，突出抓好流动人口等薄弱环节，综合治理出生人口性别比升高问题，确保人口出生率控制在12‰以内。开展新一轮土地利用总体规划修编，严格耕地、森林、水、矿产等各类资源保护，严禁小水电和矿产资源无序开发。加强地质灾害防治。继续整治城乡大气粉尘污染、河道污染、噪音污染和脏、乱、差现象，治理国省道沿线、主要水流域、主要景区、重点乡镇的“青山挂白”，建设沿路沿河绿化带、城镇公共绿地和农村生态林。

　　繁荣社会事业。落实以县为主的基础教育管理体制，加强“双高普九”工作，推进高中课程改革，全面完成中小学D级危房校舍改造，积极稳妥推进农村中小学布局调整，加强农村初中和边远山区寄宿制学校建设，解决好外来务工人员子女就学问题。加快龙岩学院、闽西职业技术学院、龙岩电大等高校建设。加强职业技术教育。实施文化精品工程，依法保护和加快开发闽西汉剧等文化遗产，加强文化市场监管，保障文化产业健康发展。办好第十二届市运动会和第七届市老年人运动会，抓好第十三届省运会场馆建设，加强体育人才培养，开展全民健身运动。继续推进市级教育、文化、卫生、体育以及市、区两级社会事业资源整合。

　　加强就业服务。做好大中专毕业生就业和军转安置服务，加快劳动力市场建设，加大劳动执法力度，巩固治理拖欠农民工工资成果，维护劳动者合法权益。全面落实再就业优惠政策，加强再就业培训，完善再就业资金筹措机制和小额贷款担保机制。继续推进劳务派遣工作。

　　提高社会保障水平。积极推进养老、失业、医疗、工伤、生育保险扩面征缴，提高非公企业职工参保率。对“五老”人员实行医疗补助及就医优待。继续做好农村低保工作，适当提高城市低保补助标准。抓好失地农民用征地补偿费办理养老保险试点，健全街道社区劳动保障服务组织。鼓励发展老年公寓等社会福利事业和慈善事业。

　　维护社会安定稳定。完成县级疾病预防控制、卫生监督体制改革。建立突发公共卫生事件应急体系，加强职业病监测预防和禽流感等动物疫病防范。严格化妆品、食品和医疗市场管理，继续治理“餐桌污染”，建设食品放心工程。加强社会治安综合治理，完善处置群体性事件工作预案，认真做好市领导接待日、群众来信来访及其它矛盾纠纷排查调处工作，全面推进创建“平安龙岩”活动，严厉打击各种刑事犯罪，切实解决“六合彩”赌博等治安突出问题。继续开展精神文明创建活动，贯彻落实《公民道德建设实施纲要》，实施“四五”普法规划，加强公民法制教育和社会公德、职业道德、家庭美德教育，普及科技知识。严格落实安全生产责任制，开展“安全生产基层基础建设年”活动，加强安监机构建设，强化监察执法，落实“三同时”制度，抓好隐患整改。加强国防动员、“双拥”、人防、民兵预备役等工作，提高应对突发事件和抢险施救能力。

　　同时，认真抓好统计和经济普查、民族宗教、气象、水文、档案、地方志、妇女儿童、残疾人、为民办实事等各项工作。

　　三、坚持不懈建设对人民高度负责的政府

　　全面落实科学发展观，加强政府行政能力建设。坚持以人为本，为民执政、科学理政、依法行政、从严治政，增强构建社会主义和谐社会的能力，提高驾驭经济社会发展、应对复杂局面、处置突发事件、保一方平安的水平。

　　增强发展观念，奋力开拓进取。牢牢把握发展第一要务，坚持发展为大、发展为重、发展为先，在发展中维护和实现最广大人民群众的根本利益，通过发展解决前进中的困难问题，以发展的实效体现对人民、对组织负责。增强责任意识，认真履行政府职责、部门职责和岗位职责，对工作中的困难问题，不回避、不推诿，勇于负责，敢于碰硬，善于突破。增强大局意识，自觉在发展全局中履行职能，在整体工作中找准位置，在参与发展、服务发展、推动发展中发挥作用、展示水平、体现价值。增强创新意识，认清新形势，把握新机遇，适应新要求，不断解放思想、与时俱进、开阔思路、拓展空间。增强科学发展观念，不断提高专业知识和领导水平，提高组织经济社会发展和社会管理的能力，努力实现更快更好的发展。

　　增强服务观念，提高政府效能。在全市政府系统全面开展保持共产党员先进性教育活动。加强行政服务中心、项目中心、外商投资企业服务中心、效能办、市长信箱和市长热线等服务窗口建设，畅通政企、政群联系渠道。坚持经济形势分析会、联席会、协调会、领导小组成员会议制度，完善政府部门工作运行机制，加强部门配合与联动服务。继续推行集中服务、代办服务、承诺服务。强化政府和政府部门的督促落实职能，建立有效的落实机制。健全约束和激励机制，改进目标管理考评，建立市直机关投资与发展环境评估评议制度，开展政府及其部门绩效评估试点工作，加强行政效能监察和效能投诉处理工作。抓好新一轮政府机构改革的落实到位，推进事业单位机构改革，由财政核拨和拨补的事业编制精简10%以上。

　　增强法制观念，推进依法行政。全面贯彻落实《行政许可法》、国务院《全面推进依法行政实施纲要》和省政府的实施意见。坚持民主集中制，加强民主决策、科学决策。依法规范出让国有资产，对国有资产产权转让、经营性土地出让、重大项目确定、大笔资金分配，坚持由同级政府领导班子集体决策。认真执行政府重大决策专家咨询制度、合法性论证制度、重大事项社会公示和听证制度以及社情民意反映制度，认真听取人大代表、政协委员和社会各界人士的建议意见，加强与工会、共青团、妇联、科协等人民团体的沟通联系。严格按照法定权限和程序行使权力、履行职责，落实行政执法责任制和执法过错责任追究制，开展相对集中行政处罚权试点。主动接受人大及其常委会的法律监督、工作监督，政协、民主党派、工商联、无党派人士和人民团体的民主监督，以及司法监督、舆论监督、群众监督。加强行政复议工作。

　　增强自律观念，加强廉政建设。坚持标本兼治、综合治理、惩防并举、注重预防的方针，加快健全教育、制度、监督并重的惩治和预防腐败体系。加强思想政治教育，牢记“两个务必”。严格单位内部车辆、会议、电话、公务外出等各项管理制度，降低行政成本。推进各部门政府资产的统一管理经营。增强廉洁从政意识，加强政务公开，推进透明行政。加强审计监督，强化财政性资金、预算外资金和领导干部经济责任审计。落实廉洁从政各项规定，严格执行党风廉政建设责任制，加强述廉工作，严肃查处损害群众利益和违法违纪的人和事。

　　各位代表，龙岩已经进入新一轮加快发展的重要阶段。让我们紧密团结在以胡锦涛同志为总书记的党中央周围，在市委的领导下，高举邓小平理论伟大旗帜，全面贯彻“三个代表”重要思想，紧紧依靠全市人民，以更加坚定的信心、更加振奋的精神，更加务实的作风，鼓足干劲，奋发进取，为建设闽粤赣边联结沿海、拓展腹地的生态型经济枢纽，使闽西成为海峡西岸经济区的重要增长极而努力奋斗！
